# The Impact of Wastewater on Antimicrobial Resistance: A Scoping Review of Transmission Pathways and Contributing Factors

**DOI:** 10.3390/antibiotics14020131

**Published:** 2025-01-26

**Authors:** Maria Clara La Rosa, Andrea Maugeri, Giuliana Favara, Claudia La Mastra, Roberta Magnano San Lio, Martina Barchitta, Antonella Agodi

**Affiliations:** Department of Medical and Surgical Sciences and Advanced Technologies “GF Ingrassia”, University of Catania, 95123 Catania, Italy; mariclalarosa@gmail.com (M.C.L.R.); andrea.maugeri@unict.it (A.M.); giuliana.favara@unict.it (G.F.); claudia.lamastra@unict.it (C.L.M.); robertamagnanosanlio@unict.it (R.M.S.L.); martina.barchitta@unict.it (M.B.)

**Keywords:** antibiotic-resistant bacteria (ARB), antibiotic resistance genes (ARGs), antimicrobial resistance (AMR), wastewater treatment plants (WWTPs), public health

## Abstract

Background/Objectives: Antimicrobial resistance (AMR) is a global issue driven by the overuse of antibiotics in healthcare, agriculture, and veterinary settings. Wastewater and treatment plants (WWTPs) act as reservoirs for antibiotic-resistant bacteria (ARB) and antibiotic resistance genes (ARGs). The One Health approach emphasizes the interconnectedness of human, animal, and environmental health in addressing AMR. This scoping review analyzes wastewater’s role in the AMR spread, identifies influencing factors, and highlights research gaps to guide interventions. Methods: This scoping review followed the PRISMA-ScR guidelines. A comprehensive literature search was conducted across the PubMed and Web of Science databases for articles published up to June 2024, supplemented by manual reference checks. The review focused on wastewater as a source of AMR, including hospital effluents, industrial and urban sewage, and agricultural runoff. Screening and selection were independently performed by two reviewers, with conflicts resolved by a third. Results: Of 3367 studies identified, 70 met the inclusion criteria. The findings indicated that antibiotic residues, heavy metals, and microbial interactions in wastewater are key drivers of AMR development. Although WWTPs aim to reduce contaminants, they often create conditions conducive to horizontal gene transfer, amplifying resistance. Promising interventions, such as advanced treatment methods and regulatory measures, exist but require further research and implementation. Conclusions: Wastewater plays a pivotal role in AMR dissemination. Targeted interventions in wastewater management are essential to mitigate AMR risks. Future studies should prioritize understanding AMR dynamics in wastewater ecosystems and evaluating scalable mitigation strategies to support global health efforts.

## 1. Introduction

Antimicrobial resistance (AMR) has become a critical global issue in the 21st century, affecting human health, animal populations, and environmental ecosystems [1,2]. The overuse and misuse of antibiotics in healthcare, veterinary practices, and agriculture are major contributors to the rapid escalation of AMR worldwide. Factors such as the unregulated sale of antibiotics without prescriptions, inadequate sanitation practices, and the release of unmetabolized antibiotics or their residues into the environment through fecal matter, manure, and industrial waste have exacerbated this problem. Therefore, environments that manage human waste, such as composting toilets, and sectors like livestock and aquaculture farms are recognized as significant hotspots for the emergence and spread of antibiotic-resistant bacteria (ARB) [3].

In particular, municipal sewage systems and wastewater treatment plants (WWTPs) play a crucial role as both reservoirs and pathways for the dissemination of antibiotic resistance. Wastewater, often contaminated with antibiotic residues, nutrients, resistant microorganisms, and antibiotic resistance genes (ARGs), provides an ideal environment for the emergence of multidrug resistance among environmental bacteria. Specifically, WWTPs are abundant in both organic and inorganic nutrients. They provide favorable conditions, such as an optimal temperature and pH, which facilitate the proliferation of ARB. Moreover, the close proximity of cells in WWTPs fosters individual cell–cell interactions, contributing to the dissemination of ARB and, ultimately, the evolution of ARGs [4,5,6]. It also facilitates the horizontal transfer of resistance genes, which exacerbates the spread of AMR [7,8,9]. Horizontal gene transfer (HGT) enables bacteria to share genetic material through three primary mechanisms: conjugation, transformation, and transduction. These processes are driven by mobile genetic elements (MGEs) like plasmids, transposons, and integrons [10,11]. In addition, ineffective wastewater treatment processes can lead to the accumulation of ARB, which then spread through direct contact with water, the irrigation of agricultural land, the water supply, and the contamination of aquatic ecosystems [4,7,12]. This complex interplay between human activity and microbial evolution presents a significant challenge to public health and environmental safety, as these facilities and environments create conditions that promote the proliferation and spread of antibiotic-resistant microorganisms, intensifying the broader public health crisis [13,14,15]. Therefore, the fight against antibiotic resistance necessitates an approach that takes into account both environmental and human health concerns, concentrating on antibiotic pollution in the environment. This approach offers a comprehensive viewpoint and substantial empirical evidence regarding the link between environmental antibiotics and their impact. The One Health perspective, advocated by the World Health Organization (WHO), envisions human health issues, including AMR, within the interconnected context of humans, animals, and the environment [16]. Gaining insights into the transmission of ARGs from WWTPs and their contribution to resistance dissemination is essential for formulating effective measures to control the spread of antibiotic resistance in the environment. This scoping review aimed to systematically analyze and map the existing literature on the role of wastewater and WWTPs in the spread of AMR. It sought to identify and categorize the primary pathways through which AMR disseminates via wastewater and WWTPs, examine the environmental, biological, and chemical factors that drive this spread, and highlight gaps in the literature to inform future research and policy development. This review addressed several key research questions: (i) What are the main pathways through which AMR spreads via wastewater and WWTPs? (ii) How do environmental, biological, and chemical factors influence the AMR spread in these systems? (iii) What interventions or strategies have been explored or proposed to mitigate the impact of wastewater on AMR transmission? By providing a comprehensive overview of how wastewater contributes to the AMR spread and identifying critical influencing factors, this review aimed to enhance the understanding of the issue and guide public health initiatives and environmental management practices designed to reduce AMR transmission.

## 2. Results

### 2.1. Descriptiono of Study Selection

**Figure 1:**
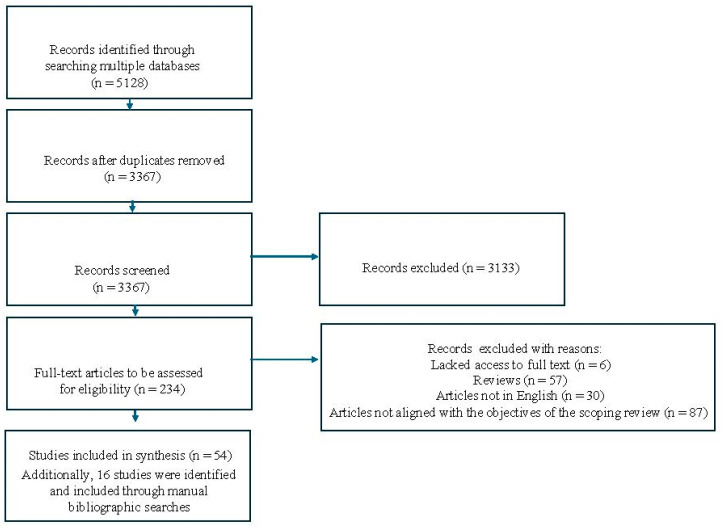
Selection of studies included in the scoping review.

After removing duplicates, a total of 3367 articles were identified through the literature search. During the initial screening of titles and abstracts, 3133 articles were excluded, leaving 234 full-text articles for a more in-depth evaluation. Of these, 180 were excluded for the following reasons: 6 lacked access to the full text, 57 were reviews, 30 were not in English, and 87 were not aligned with the objectives of this scoping review. As a result, 54 studies were deemed eligible and included in this scoping review. Additionally, 16 studies were identified and included through manual bibliographic searches (Figure 1).

In the following sections, we summarize the key studies investigating the role of wastewater in the spread of AMR. We begin by examining the presence of antibiotics in wastewater, highlighting how antibiotic residues and heavy metals contribute to the development and amplification of resistance. Next, we analyze the role of microbial communities in promoting AMR dissemination. We then evaluate how various wastewater treatment methods and water quality parameters either curb or facilitate the spread of AMR. Finally, we outline the primary mechanisms through which ARGs are transferred from environmental reservoirs to humans, emphasizing the critical pathways involved in this process.

### 2.2. Aquatic Environments as Reservoirs of Antimicrobial Resistance: The Critical Role of Wastewater in AMR Dissemination

The global spread of AMR through various environmental reservoirs poses a critical challenge to public health. Numerous studies have analyzed the role of wastewater and aquatic environments in disseminating ARGs. For example, Varela et al. (2016) investigated the spread of AMR in wastewater using *Aeromonas species* as model organisms [17]. These bacteria, capable of harboring beta-lactam ARGs (e.g., *blaOXA*,) and plasmid-mediated quinolone resistance (*PMQR*) (*qnrS*2; *aac(*6*′)-Ib-cr*) mechanisms, have demonstrated a high capacity to propagate resistance through HGT. The majority of isolates were identified as belonging to the species *Aeromonas caviae* (50%) and *Aeromonas hydrophila* (41%). The *aac(*6*′)-Ib-cr* and *blaOXA* genes were detected in 58% and 56% of the tested isolates, respectively. The study isolated 112 quinolone-resistant *Aeromonas* strains from diverse wastewater sources, including hospital wastewater (HWW), untreated urban wastewater (UWW), and treated wastewater (TWW). Genetic analyses revealed a high prevalence of the *qnrS*2 gene, associated with *IncU*-type plasmids, primarily in urban wastewater, while it was rare in HWW, suggesting a non-clinical origin. Conversely, the *aac(*6*′)-Ib-cr* gene (a gene conferring resistance to quinolones via plasmid-mediated mechanisms) was detected across all water types, including TWW, highlighting the resilience of *Aeromonas* during treatment processes [17]. Similarly, Güneri et al. (2022) identified fosfomycin-resistant (*fosR*) *Enterobacterales* in both hospital and municipal wastewater [18]. Their study emphasized how selective pressures from antimicrobials and biocides contribute to the persistence of these bacteria in water. Critical pathogens such as *ESKAPE* (*Enterococcus faecium*, *Staphylococcus aureus*, *Klebsiella pneumoniae*, *Acinetobacter baumannii*, *Pseudomonas aeruginosa*, and *Enterobacter* spp.) and *E. coli*, resistant to multiple first-line and last-resort antibiotics, were found to adapt rapidly through the exchange of MGEs. ARGs such as *fosA*3 and *fosA*4 were particularly prevalent in hospital wastewater, underscoring the role of human HWW as a significant reservoir of AMR and the urgent need for stricter wastewater management to mitigate public health risks [18]. Dropa et al. (2024) highlighted that HWW represents one of the most concerning sources of ARGs [19]. Their study analyzed four wastewater sources: a WWTP (including raw wastewater, secondary effluents, and reused water), HWW, veterinary hospital wastewater (VH), and wastewater from a pig farm (PF). Qualitative analyses detected ARGs across all antibiotic classes studied. Clinically significant genes were identified, including beta-lactamase genes with an extended spectrum *(CTX-M*) and *AmpC* (*CMY*), carbapenemase genes (*KPC*, *OXA-*48, *IMP*, *VIM*, *NDM*), enzymes modifying fluoroquinolones and aminoglycosides (*aac(*6*′)-Ib-cr*), transferable fluoroquinolone resistance proteins (*qnr*, *OqxAB*), colistin resistance phosphoethanolamine transferase genes (*mcr-*1 and *mcr-*3), and RNA methylase genes (*rmtD*). At all sites, genes coding for class 1 integrons, CTX-M (groups 1, 2, and 9), *qnrB*, *qnrS*, *OqxAB*, and *mcr-*3 were detectable. Genes such as *blaCTX-M-*8, *blaCMY*, *blaKPC*, *blaOXA-*48, *qnrD*, and *mcr-*1 were found at over 75% of the sampling sites. Less common genes, such as *rmtD* and *rmtG*, were found at 65% of the sites, while *mcr-*2 was exclusively detected at the PF. Genes coding for carbapenemase, especially *blaKPC*, were present at all sites. In the VH, the highest frequencies were found for class 1 integrons and genes coding for *CTX-M* and *qnr*. In HWW, the frequency of genes like *blaKPC* reached 91.7%. HWW showed a high frequency of various genes, including those causing resistance to beta-lactam antibiotics, fluoroquinolones, and aminoglycosides and genes responsible for polymyxin resistance. Notably, the qualitative screening identified 23 ARGs, 16 of which were detected at frequencies exceeding 75% [19]. Therefore, aquatic environments, especially HWW, play a critical role in the global cycle of AMR. These findings underscore the critical role of wastewater as a key reservoir of AMR, facilitating the widespread dissemination of ARGs across various environmental contexts. Studies such as those by Varela, Güneri, and Dropa highlight the urgent need for strategies to limit the spread of AMR, starting with improved wastewater treatment and the improved control of contamination sources. In addition, Szczepanowski et al. (2009) discovered that bacteria in WWTPs harbor a wide range of ARGs, including some recently identified in clinical settings [20]. This indicates active genetic exchange between clinical and WWTP bacteria. Furthermore, the purification processes in the analyzed WWTP did not effectively reduce the variety of detectable ARGs in the final effluents. The identification of approximately 64% of the 192 reference ARGs in bacteria from the WWTP’s final effluents suggests a potential risk of these resistance determinants spreading to downstream environments, potentially facilitating their dissemination among environmental bacteria [20]. In this context, in February 2022, Odih et al. made a notable discovery [21]. They studied the presence of carbapenem-resistant Acinetobacter baumannii in raw and treated HWW and analyzed their phylogenetic relationships with clinical isolates of the same bacterium. During their investigation, they identified two bacterial isolates in treated HWW that were almost identical to two clinical isolates obtained from a patient admitted to the same facility three months later, in May 2022. These four sequence types in ST919 strains of *Acinetobacter baumannii*—two from wastewater and two from the patient—were clonal, exhibiting only four to nine single-nucleotide polymorphism (SNP) differences, and all harbored both the *blaNDM-*1 and *blaOXA-*23 ARGs. Interestingly, *blaNDM-*1 and *blaOXA-*23 are critical genes that encode enzymes responsible for resistance to carbapenems, one of the last-resort antibiotics used to treat *Acinetobacter baumannii*. The spread of these ARGs significantly limits the treatment options available, making infections harder to control and treat effectively. Although the current data do not clarify the direction or link of transmission between these strains, the findings suggest a probable transmission link between the hospital environment and the human population. The close genetic relationships and clustering patterns observed between the clinical and wastewater isolates, even in the final treated effluent, point to possible transmission chains from the hospital environment—or even directly from patients—to the surrounding environment that receives the TWW. Additionally, the presence of carbapenemase genes, which were significantly associated with the presence of multiple AMR determinants, was remarkably high among the wastewater isolates. This emphasizes the potential role of HWW as a reservoir and a conduit for the spread of highly ARB strains [21]. Khanal et al. revealed the widespread presence and spread of AMR in the environment, emphasizing the urgent need for effective strategies to reduce and control the spread of antibiotic resistance [22]. In particular, the prevalence of *extended-spectrum beta-lactamase* (ESBL)-producing bacteria and their associated ARGs was assessed in wastewater and river water. A total of 35 wastewater samples were collected from various sources, including pharmaceutical industries, hospitals, municipal sewage systems, and rivers. Among the isolated bacteria, 57.72% were identified as ESBL producers, with *Escherichia coli* (40.85%) and *Klebsiella pneumoniae* (32.39%) being the most common. The analyses revealed that municipal and hospital wastewater contained a higher density of ARB compared to pharmaceutical wastewater and significantly contributed to the contamination of nearby watercourses. Furthermore, plasmids and MGEs, such as IS903 and IS26, were identified, which facilitate the dissemination of antibiotic resistance. However, while the incidence of ESBL-producing bacteria was significantly reduced in treated effluents from hospital and municipal WWTPs, a comparable level of reduction was not observed in treated effluents from pharmaceutical wastewater treatment facilities [22]. Therefore, industrial wastewater (IWW) also plays a significant role in the spread of AMR. Milaković et al. (2018) demonstrated that the discharge of pharmaceutical effluents significantly alters the physicochemical properties and bacterial community composition of receiving river sediments, contributing to the enrichment of macrolide ARGs and integrons [23]. Their study focused on effluents from an azithromycin manufacturing site and sediment samples collected upstream and downstream of the discharge point during both the winter and summer seasons. Pharmaceutical production effluents, which often contain high concentrations of antibiotics, are a key driver of the selection and spread of ARGs in the environment. The release of these industrial effluents not only increases the levels of macrolide antibiotics, heavy metals, and nutrients in the receiving sediments but also significantly elevates the abundance of ARGs and integrons. A primary mechanism behind this phenomenon is the dissemination of a transferable resistome, which facilitates the persistence of macrolide ARGs in downstream sediments even after the discharge of inadequately treated effluents. Notably, the ARGs targeted in the study were detected in sediments up to 11 km downstream of the discharge site. Among the five macrolide ARGs investigated, three sub-types—macrolide phosphotransferase (*mphG*), ribosome protection protein (*msrE*), and ribosomal methylase (*ermB*)—were predominant in the effluents and exhibited a marked increase in abundance within exposed sediments. Although the *mefC* and *mphE* genes, encoding efflux and another macrolide phosphotransferase, were less prevalent in the effluents compared to *msrE*, *mphG*, and *ermB*, their abundance also significantly increased in sediments impacted by the discharge. These findings emphasize the substantial environmental impact of pharmaceutical effluents on the dissemination and persistence of antibiotic resistance in aquatic ecosystems [23]. Marathe NP et al. highlighted how WWTPs serving pharmaceutical industries that produce antibiotics can serve as a fertile ground for gene transfer events due to the high bacterial densities and strong, persistent selective pressures from antibiotic-contaminated waste [24]. Using a bacterial isolation-based approach, various samples were collected from different stages of the wastewater treatment process at the PETL treatment plant in Patancheru, near Hyderabad, India. The samples included were taken from influent water, aeration tanks, sedimentation tanks, and different types of sludge (secondary sludge, dewatered sludge, and old dried sludge). The analysis revealed the presence of a wide range of multi-resistant bacteria, with the majority (86%) resistant to 20 or more antibiotics from 12 different classes. Opportunistic bacteria such as *O. intermedium* and *P. rettgeri* showed particularly high resistance to drugs. The qPCR analysis of DNA showed a high prevalence of integrons (95%), particularly those belonging to class 1, indicating an ideal environment for HGT [24]. Furthermore, it was revealed that the total abundance of ARGs in the sludge from a treatment facility receiving wastewater from the pharmaceutical production of the macrolide antibiotic azithromycin was three times higher compared to that in the municipal sludge from a sewage treatment plant in Zagreb, Croatia. Specifically, the overall quantity of ARGs per 16*S rRNA* gene copy was approximately three times greater in the industrial sludge than in the municipal sludge (*p* = 3.24 × 10^−5^). A significantly higher total abundance of integrases and transposases was observed in the industrial samples (*p* = 1.32 × 10^−6^), suggesting an enrichment of mobile antibiotic ARGs. Additionally, the relative abundance of plasmids in the industrial samples was elevated (*p* = 1.95 × 10^−6^). Moreover, the diversity of detected plasmids was also greater in the industrial samples compared to the municipal ones, with an average of 1497 versus 926 plasmid types (*p* = 0.00067). The collective results imply that in environments with high levels of antibiotic contamination, the selection pressure predominantly favors taxonomic changes towards inherently resistant communities or strains carrying resistance mutations. However, the total count of distinct ARGs, known as the resistance gene richness, was lower in the industrial sludge compared to the municipal sludge (*p* = 0.00136). Significantly, although the total abundance of macrolide ARGs did not surpass that of a municipal WWTP, the presence of ARGs typically linked with MGEs like integrons was higher. This indicates that heightened antibiotic exposure leads to augmented genetic mobility within microbial communities. The absence of elevated levels of macrolide ARGs suggests that the intense selection pressure exerted by macrolide antibiotics has prompted taxonomic shifts towards inherently resistant species or strains harboring chromosomal resistance mutations, rather than the acquisition of mobile resistance determinants for macrolides [25]. Anthropogenic pollution significantly influences both the microbial community and the antimicrobial resistome. In 2024, Sivalingam et al. addressed the role of anthropogenic pollution in the dissemination of ARGs, with a particular focus on MGEs, which are considered to be crucial for the mobility of these genes [26]. The research was conducted using a dual approach of field analysis and controlled laboratory experiments, examining water from three lakes (Maggiore, Orta, Mergozzo) and effluents from three WWTPs. The WWTPs employed different disinfection methods: chlorination (Verbania) and peracetic acid (Cannobio, Gravellona Toce). Field analysis revealed that the diversity of ARGs was notably higher in TWW compared to lake water, even when considering high-risk ARGs alone. This suggests that pollution from human activities boosts the variety of ARGs in TWW. Additionally, the richness of microbial communities, the antimicrobial resistome, and high-risk ARGs was greater in TWW than in lake waters, both for intracellular (iDNA) and extracellular DNA (eDNA). However, except for high-risk ARGs, the richness of ARGs was significantly higher in iDNA compared to eDNA. Laboratory experiments further demonstrated that anthropogenic pollution positively influenced the transformation of *Gfp* plasmids carrying ARGs within a natural microbial community. The experiments mixed lake water with untreated wastewater (NTWW) in varying proportions and used plasmids carrying ARGs to kanamycin and streptomycin under selective pressure. The results showed that pollution facilitated the uptake and selection of extracellular plasmids harboring ARGs. These findings underscore the significant role of anthropogenic pollution in shaping microbial communities, enhancing the resistome, and driving the spread of antibiotic resistance through HGT mechanisms in aquatic ecosystems [26].

### 2.3. Occurrence of Antibiotics in Wastewater

Antimicrobial substances are released into the environment through human-derived wastewater from various sources, including households (domestic), hospitals (clinical), veterinary sources, and animal farming, as well as pharmaceutical factories (industrial) [27]. Indeed, studies have indicated that a significant portion of antimicrobial substances is not completely metabolized during their therapeutic use. Approximately 30–90% of these substances are believed to enter wastewater in their active forms. Consequently, various classes of antibiotics have been extensively detected in WWTP facilities and their surrounding environments globally [28,29]. In particular, antimicrobial substances and their resulting by-products have been identified in the environment at concentrations ranging from nanograms per liter (ng L-1) to micrograms per liter (µg L-1) upon their introduction into aquatic systems [30]. These substances are identified as emerging micropollutants, and a concern associated with their presence in the environment is an increase in ARGs and the development of ARB [31,32]. The level of environmental pollution is influenced by the patterns of their usage. A rise in consumption, particularly during the colder seasons when infection rates are higher, leads to an elevated presence of these substances in environmental systems [33]. This is associated with the emergence of ARB resistant to multiple antibiotics and their rapid proliferation [34].

The antibiotic resistance of environmental bacteria could be significantly impacted by the antibiotic selection pressure in the environment. For example, the emergence of AMR in a non-pathogenic environmental bacterium, *Pseudoxanthomonas mexicana*, isolated from laboratory-scale bioreactors treating wastewater containing streptomycin, was assessed. Bioreactors are employed for the biological treatment of wastewater, where biological (enzymatic) processes facilitate the breakdown of pollutants, while subsequent units separate the purified water from solids. The analysis involved two strains of *P. mexicana*: one resistant to streptomycin (SR) and one non-resistant (SNR). The analysis revealed that the SR strain showed resistance to streptomycin, ampicillin, kanamycin, and sulfamethoxazole, which was attributed to a class 1 *In-Tn*5393*c* gene array. This gene array, commonly found in pathogenic strains, is associated with resistance to multiple antibiotics and has been acquired through HGT. In this study, the *In-Tn*5393*c* array showed a unique arrangement, with the presence of *IS*6100 and *IS*256 elements, which evolved in response to the streptomycin pressure. Transcriptional and proteomic analyses showed that genes and proteins involved in antibiotic resistance (e.g., *strA*, *strB*, *aadB*) were overexpressed in the SR strain. In particular, 30S ribosomal genes (*rpsA* and *rpsU*) were highly activated, indicating a regulatory mechanism in response to streptomycin. Therefore, the pressure of streptomycin stimulated the acquisition of these ARGs, as well as the overexpression of ribosomal proteins, which are involved in protein synthesis and the stress response [35]. Voigt et al. have also investigated the associations between antibiotic residues, ARB, and ARGs in anthropogenically influenced wastewater [36]. The analysis was conducted on wastewater samples collected from 13 sampling sites, which included HWW (from high-level hospitals), mixed urban wastewater (clinical and urban influences), and non-clinically influenced wastewater, both treated and untreated, from WWTPs in urban and rural areas. A key aspect of the study was the simultaneous detection of antibiotic residues and third-generation cephalosporin-resistant *Pseudomonas aeruginosa* (3*GCR*). The data revealed a strong association between ciprofloxacin residues and the presence *of* 3*GCR P. aeruginosa*. Ciprofloxacin residues were found at higher levels in clinical wastewater samples, with concentrations ranging from 0.2 µg/L to 88.3 µg/L. In these samples, the likelihood of detecting 3*GCR P. aeruginosa* was significantly higher compared to that in non-clinically influenced urban wastewater. Specifically, the odds ratio analysis showed that in samples with ciprofloxacin residues, the probability of detecting 3*GCR P. aeruginosa* increased by a factor of 2 to 8. The positive identification of residual concentrations of carbapenems, specifically meropenem, was correlated with corresponding carbapenemases, such as *blaNDM*, *blaVIM*2, and *blaOXA*48. Additionally, notably high odds ratios were identified between antibiotics such as linezolid, vancomycin, and ampicillin and the genes *blaOXA*48, *blaCTX-M*, and *blaVIM*2 [36]. Guo et al. (2017) employed sequential batch biological reactors (SBRs) to simulate wastewater treatment conditions [37]. By introducing sulfamethoxazole and a chemical oxygen demand (COD), they investigated the impact of these contaminants on the selection of ARGs and the alteration of bacterial community structures. The presence of sulfamethoxazole and a COD led to a notable increase in ARGs, indicating that these contaminants may drive the selection of ARB. Specifically, the ARG accumulation over time in the SBR system followed a linear trend, with ARG levels gradually decreasing in the absence of a sulfamethoxazole selection pressure. Therefore, the continuous introduction of antibiotics in real-world SBR systems inevitably contributes to the development and spread of ARGs. Over a period of 55 days, the *sul I* gene concentrations increased to 4.79 × 10^5^ copies/mL, 2.10 × 10^5^ copies/mL, and 1.05 × 10^5^ copies/mL in groups exposed to 10 mg/L, 0.10 mg/L, and 0.001 mg/L of sulfamethoxazole, respectively. Additionally, significant changes were observed in the bacterial community compositions, with ARB becoming more prevalent [37]. This observation aligns with the findings of Collado et al. (2013) [38]. Their research on an SBR system revealed that the abundance of ARGs increased progressively with the duration of treatment. Notably, the lowest ARG levels were observed in the group treated with 0.001 mg/L sulfamethoxazole. This pattern indicates that higher concentrations of antibiotics tend to promote the proliferation of ARGs. Remarkably, both the sul I and sul II abundances linearly increased with the SBR operation time in the presence of sulfamethoxazole, while slightly decreasing in control groups without sulfamethoxazole. The COD value may influence the impact of sulfamethoxazole on ARGs. Insufficient COD levels may inhibit bacterial growth, thereby limiting the proliferation of sul I and sul II genes. One plausible explanation is that compounds contributing to the COD serve as nutrition for bacteria. Without an adequate nutrient source, organisms may struggle to grow normally, even under the selection pressure exerted by sulfamethoxazole. Similarly, it has been observed that the prolonged presence of the antibiotic erythromycin in wastewater, even at low concentrations, has the potential to exert selective pressure on ARB, contributing to the development of antibiotic resistance [39]. Significant and positive correlations (0.883  <  r  <  0.929, *p*  <  0.05) have been identified between the presence of the erythromycin antibiotic and the *ere(A*), *ere(B)*, and *mef(A)/mef(E*) genes. This suggests that the selection pressure on these ARGs may be linked to the presence of erythromycin, despite its very low concentration in wastewater. Additionally, a significant correlation (r  =  0.829, *p*  <  0,05) was observed between *erm(B)* genes and tetracycline (TC), while other identified erythromycin ARGs did not show such a correlation [40]. In contrast, in 2003, Ohlsen et al. conducted experiments on sewage agar plates using gentamicin, tetracycline, erythromycin, and ciprofloxacin at concentrations ranging from 0.001 to 0.1 mg/L [41]. Their aim was to evaluate how antibiotics in sewage contribute to the horizontal transmission of ARGs in Gram-positive pathogens, specifically using conjugative antibiotic resistance plasmids derived from *S. aureus*. They found that the presence of these antibiotics at a concentration of 0.1 mg/L did not significantly impact the transfer rates. The results suggest that while resistance gene transfer in Gram-positive pathogens can occur in environmental settings, the introduction of antibiotics into sewage does not enhance the spread of conjugative antibiotic resistance plasmids through increased transfer rates. This is particularly true for the low-level concentrations of antibiotics typically found in the environment [41]. Xu et al. examined the effects of CIP and a dialkyldimethyl ammonium compound (DADMAC) on nitrifying systems using three SBRs (R1, R2, R3) operated with sludge from WWTPs in Beijing, China, over a period of 150 days [42]. CIP had a minimal impact on the ammonia oxidation, while DADMAC, especially at higher concentrations, significantly inhibited it. Their combination had a stronger inhibitory effect and caused partial nitrification, with the highest nitrite accumulation in R3. Additionally, the mixture of CIP and DADMAC increased the size of sludge particles and boosted the abundance and transfer of ARGs, promoting their spread in the system. The findings suggest that while these chemicals can control bacterial activity, they also enhance the dissemination of ARGs [42]. In 2019, it was found that the primary mechanism driving the emergence of tetracycline ARGs under prolonged exposure to low levels of tetracycline stress was HGT. The increase in tetracycline concentrations rapidly drove the evolution of tetracycline resistance. Specifically, at concentrations of 20 and 50 μg/L, the efflux pump became the primary resistance mechanism. Notably, *tet(A)* and *tet(G*) showed positive correlations with class 1 integrons (*p* < 0.05). Consequently, prolonged exposure to low levels of tetracycline tended to increase the relative abundance of class 1 integrons as a defensive response, potentially further promoting the prevalence of efflux pump genes [43]. Brown et al. confirmed that the varying antibiotic levels in sewage are linked to the HGT of ARGs, as well as to changes in microbial communities and the diversity of resistance gene behavior within activated sludge systems [44]. Their findings indicated that the impact of antibiotics on the selection of ARB is influenced by the existing interactions within the microbial community and may be facilitated by HGT [44]. Sutradhar et al. conducted research to address the lack of a quantitative understanding regarding how antibiotics interact in environments with a constant flow [45]. They focused on experimentally monitoring populations of *E. coli* exposed to subinhibitory concentrations of antibiotic combinations with different interaction types: synergistic, antagonistic, and additive. Their findings revealed that *E. coli* populations subjected to synergistic and antagonistic antibiotic conditions exhibited behaviors that deviated significantly from the expected outcomes. Specifically, *E. coli* populations exposed to synergistically interacting antibiotics developed less resistance than predicted, suggesting that these combinations might suppress resistance development. Conversely, *E. coli* populations exposed to antagonistically interacting antibiotics showed resistance development that was dependent on the specific ratio of antibiotics used. This implies that not only the type of antibiotic interaction but also the relative concentrations of the antibiotics play a crucial role in predicting how resistance develops [45]. It was also observed that the tetracycline concentration gradient influenced the development patterns of tet genes under anaerobic conditions. In the anaerobic hybrid reactor (AHR), the abundance of intI1 genes increased with rising tetracycline dosages, while they decreased in the completely mixed aerobic reactor (CMAR). In the AHR, the dosage of tetracycline showed significant positive correlations with *tet* genes such as *tet(C*), *tet(G*), *tet(X)*, *tet(O)*, and *tet(M)* (r = 0.904, 0.446, 0.649, 0.573, and 0.898, respectively; *p* < 0.05). Conversely, a significant negative correlation was found between the dosage of tetracycline and *tet(W*) (r= −0.667, *p* < 0.01), indicating that tetracycline exerted selection pressure on the bacterial hosts carrying these tet genes. In the CMAR, the dosage of tetracycline exhibited significant correlations with *tet(A)*, *tet(C)*, *tet(G)*, *tet(X)*, and *tet(O)* (r = −0.415, 0.583, 0.790, 0.746, and −0.767, respectively; *p* < 0.05). These findings suggest a close relationship between the presence of tetracycline and the occurrence of tet genes. Additionally, a notable correlation between the dosage of tetracycline and intI1 genes was found in both the AHR (r = 0.698, *p* < 0.01) and CMAR (r = −0.477, *p* < 0.05). This suggests that the presence of tetracycline in wastewater may exert selection pressures for bacterial resistance and influence the evolutionary pathways of tet genes mediated by intI1 genes. The variations in the gene distribution between the AHR and CMAR could be due to differences in the bacterial growth rates, oxygen availability, and nutrient levels. Moreover, strong correlations were observed between intI1 genes and tet genes in anaerobic–aerobic sequential (AAS) bioreactors [e.g., *tet(C)*, *tet(G)*, *tet(X)*, *tet(O)*, and *tet(M)* in the AHR*; tet(A)*, *tet(W)*, *tet(O)*, and *tet(M)* in the CMAR], highlighting their significant role in the horizontal spread of tetracycline resistance. The AAS system could be a viable and cost-effective alternative for simultaneously reducing nutrients, antibiotics, and antibiotic resistance in practical applications [46].

### 2.4. The Role of Antibacterial Residues and Heavy Metals in Promoting the Development of Antibiotic Resistance

Antibiotic resistance can arise even in the absence of direct antibiotic pressure in the environment. Studies have shown that not only antibiotics but also other factors, such as antibacterial residues and heavy metals, can contribute significantly to the development and proliferation of antibiotic resistance [47]. In contrast to antibiotics, heavy metals are not subject to degradation. Consequently, they serve as a persistent and prolonged selective force for the sustenance and amplification of antibiotic resistance. Zinc (Zn) exhibited a significant correlation with erythromycin ARGs, including the *ere(A)*, *ere(B)*, *mef(A)/mef(E)*, and *erm(B)* genes (0.823  <  r  <  0.866, *p*  <  0.05). In contrast, the correlations between chromium (Cr) and erythromycin ARGs were weaker (−0.192  <  r  <  0.582, *p*  >  0.05). Additionally, similar patterns (0.852  <  r  <  0.871, *p*  <  0.05) were observed between lead (Pb) and specific ARGs, namely the *ere(B)* and *mef(A)/mef(E)* genes. However, no significant correlation was identified between copper (Cu) and the detected erythromycin ARGs. Furthermore, there are noteworthy concerns about the robust correlations between a non-therapeutic antibacterial agent, triclosan (TCS), and erythromycin-ARGs, including the *ere(A)*, *ere(B)*, *mef(A)/mef(E)*, and *erm(B)* genes (0.859  <  r  <  0.956, *p*  <  0.05). This suggests that the presence of TCS in wastewater holds significant importance as a selecting agent influencing the evolution of erythromycin ARGs [40]. Research has indicated that AMR in wastewater can respond to regulatory changes. The study showed that the cross-resistance to clinically significant antibiotics in bacteria, influenced by non-therapeutic antimicrobials, varies depending on the type of antimicrobial used, the bacterial taxonomy, and temporal factors. This highlights the complexity of AMR patterns and underscores the need for nuanced regulatory approaches to manage and mitigate the spread of resistance in diverse microbial communities. Notably, the study identified numerous clinically relevant bacteria, including opportunistic pathogens capable of causing multidrug-resistant (MDR) infections. The research underscored significant differences in the taxa and cross-resistance patterns influenced by various antimicrobials within a community relevant to the human microbiome. Although bacteria resistant to benzalkonium chloride (BC) currently represent a small portion of the culturable community, the extensive use of BC could lead to an increased proportion of resistant microorganisms. A key finding has been the clear association between BC resistance and cross-resistance to colistin, a last-resort antibiotic, indicating a need for further investigation. As BC replaces TCS in many consumer products, understanding its impact on the spread of resistance to clinically important antibiotics is crucial, particularly concerning the cross-resistance to critical, last-resort treatments [48]. Moreover, microorganisms also play a crucial role in the HGT of ARGs. A metagenomic approach was utilized to simultaneously identify ARGs and antibacterial biocide and metal resistance genes (BMRGs), along with their corresponding microbial hosts with high mobility during the aerobic granular sludge (AGS) formation process. The results indicated that the relative abundance of BMRGs was 88–123 times higher than that of ARGs. The AGS formation process more readily enriched BMRGs, posing a greater risk of drug resistance from BMRGs compared to ARGs. The enrichment of ARGs and BMRGs in AGS was closely linked to several enhanced microbial metabolisms, including the cell motility, transposase activity, and ATP-binding cassette transporters, along with their regulatory genes [49].

### 2.5. Wastewater Biofilms as Key Players in the Amplification and Transmission of AMR

Studying microbial communities in contaminated environments, such as wastewater, reveals crucial information regarding the fate of ARGs and related issues (e.g., AMR; ARB) Auguet et al. (2017) analyzed wastewater and biofilm samples collected at the inlet and outlet of a pressurized sewer pipe, highlighting the role of sewer biofilms as sources and reservoirs of ARB [50]. They found that the composition of the bacterial community, rather than the concentration of antibiotics, was the main factor driving the diversity of the resistome in sewers. Specifically, they observed a higher abundance of bacteria with a high sequence identity (>98%) to well-known human pathogens in biofilms collected at the inlet of the sewer pipe, suggesting that the composition of the bacterial community favored the presence of ARB [50]. Brienza et al. conducted a laboratory experimental study using reactors that simulated irrigation systems [51]. The research focused on the role of biofilms in antibiotic degradation and the dissemination of ARGs. Polyethylene disks were submerged in TWW to facilitate biofilm growth. These biofilms, composed of microbial communities, were exposed to six antibiotics widely detected in wastewater: amoxicillin, ofloxacin, sulfamethoxazole, trimethoprim, clarithromycin, and cephalexin. This exposure created an environment highly conducive to the selection and proliferation of AMR. The findings highlighted the dual role of biofilms: while they reduce the ARB in wastewater, they also act as reservoirs for ARGs. In fact, the degradation process was influenced more by the bacterial community composition than by the direct presence of antibiotics. Exposure to antibiotics did not significantly impact the biofilm thickness or the structure of planktonic bacterial communities. However, biofilms served as reservoirs for ARB, potentially reintroducing resistance into less diverse wastewater ecosystems. ARGs such as *sul1*, *ermB*, and *intl1* were found to be prevalent in both biofilms and wastewater [51]. Maheshwari et al. investigated AMR and HGT in bacteria isolated from HWW [52]. They conducted an experimental study to examine the transferability of the *blaCTX-M* gene among enteric bacteria that produced ESBL in biofilms. Seventy-five percent of the ESBL-producing strains displayed moderate to strong biofilm-forming capabilities, which provided protection against antibiotics. These biofilms formed by ESBL-producing bacteria facilitated the spread of ARGs, posing a significant threat to public health. In particular, they substantially increased resistance, with minimum bactericidal concentrations (MBCs) that were 4–256 times higher compared to those of planktonic bacteria. The *blaCTX-M* gene was transferred in vitro at higher frequencies in biofilms than in planktonic bacteria (up to 9.15 × 10^−1^ transconjugants per recipient cell), as was clearly demonstrated (*p* = 0.0346) [52].

### 2.6. Treatment Methods in WWTPs and Spread of ARGs and AMR

WWTP processes play a crucial role in mitigating the environmental and public health risks associated with AMR. However, the efficiency of these systems in managing AMR varies widely depending on the treatment technologies employed, as some processes may inadvertently contribute to the selection and proliferation of resistant microorganisms (Table 1). Understanding the interplay between wastewater treatment mechanisms and the dynamics of AMR is essential for developing strategies to limit its spread and safeguard the water quality.

Therefore, some treatments inadvertently favor the survival and propagation of highly resistant strains due to selective pressures, such as exposure to residual antibiotics or sublethal disinfection doses. This is a significant limitation of current treatment technologies. Guo et al., under laboratory conditions, found that even a UV dose of 1 mJ/cm^2^ can significantly decrease the conjugative transfer frequency of the surviving bacteria [70]. However, they demonstrated that bacteria exposed to low UV doses can maintain their transfer ability even after reactivation, either through dark repair or photoreactivation. Conversely, after the application of higher UV disinfection doses, the number of reactivated cells was lower, and their transfer ability was significantly reduced [70].

Furthermore, despite reductions in the bacterial abundance, ARGs often remain detectable, particularly on MGEs, enabling their dissemination posttreatment. Indeed, effluents from TWW frequently contain transcriptionally active ARGs, which can integrate into environmental bacterial communities, perpetuating resistance. For example, Wang et al. [71]. further highlighted that WWTPs were ineffective at removing ARGs, as resistance determinants such as *intI*1, *floR*, *sul*1, and *ermB* were still detected in TWW samples. The integrase genes *intI*1 and *intI*2 were found in all samples, underscoring the role of integrons in the dissemination of ARGs. Additionally, they observed that oxygen, polyaluminum chloride (PAC), polyacrylamide, and UV disinfection significantly altered the bacterial structure in water samples. These treatments caused a shift from Gram-negative to Gram-positive bacteria, although the total biomass remained largely unchanged. While the WWTPs reduced the antibiotic content, the removal was not uniform across antibiotics: ciprofloxacin, sulfamethoxazole, and erythromycin were completely removed, whereas cephalexin was not [71]. Wastewater is often used for irrigation to address water scarcity in agriculture. However, the accumulation of antibiotics, ARB, and ARGs in effluents and soils remains an urgent and emerging concern, often attributed to the inefficiency of conventional WWTPs. In this context, effective Nature-Based Solutions (NBSs) should be prioritized for wastewater remediation. Gentile et al. conducted a pilot-scale study demonstrating that Constructed Wetlands (CWs) in various configurations are sustainable, cost-effective alternatives for wastewater treatment [72]. Beyond effectively removing traditional contaminants such as the COD and nitrogen compounds, CWs also achieved significant reductions in AMR. When comparing two pilot CW configurations—Vertical Constructed Wetlands (VCWs) and Horizontal Constructed Wetlands (HCWs)—the HCW configuration proved superior in removing wastewater contaminants, particularly the total nitrogen. The HCW’s higher efficiency stems from its innovative combination of two hydraulic flow types in series: Vertical Flow (VF) and Horizontal Subsurface Flow (HSSF). This dual-system approach harnesses the complementary biological and physical mechanisms of each flow type, enabling reclaimed effluents to meet Italian regulatory standards for reuse while reducing the AMR levels by up to 98%. Additionally, reclaimed CW and CW + UV effluents used for lettuce irrigation revealed a minimal ARB presence in the rhizosphere only, with no detectable AMR in the edible parts of the plants. This is a groundbreaking finding, showcasing the potential of CW systems to mitigate environmental and health risks associated with the global spread of AMR. Despite these promising results, further research is necessary to elucidate the fate and removal mechanisms of emerging contaminants within CWs. Full-scale experiments are also essential to assess the scalability of these findings and the feasibility of using reclaimed wastewater on large agricultural areas [72].

Real-world and laboratory data highlight that wastewater treatments, while providing a significant contribution to managing AMR, also pose challenges that cannot be overlooked. Based on the above, it is clear that implementing standardized protocols for monitoring ARGs and ARB throughout the treatment process is essential. Such protocols would offer well-defined benchmarks, enabling a more accurate evaluation of the treatment efficiency.

### 2.7. The Interaction Between Water Quality Parameters and the Spread of ARGs in Treatment Plants

Certain water parameters may serve as drivers and play a crucial role in the persistence and proliferation of ARGs in a wastewater treatment plant. Factors such as the conductivity, pH, heterotrophic plate count (HPC), total nitrogen (TN), ammonium, and chemical oxygen demand (CODMn) showed significant correlations with certain eARGs (e.g., *ampC, catA*1*, catG*) during the disinfection process. The water temperature did not show any correlations with variations in the concentration of the total observed eARGs (*p* > 0.05, *n* = 3), but a significant increase in the eARG abundance was noted when the water temperature ranged between 25 °C and 27 °C (*p* < 0.01, *n* = 3). Moreover, a much greater increase in the median concentration of the total iARGs was observed when the water temperature in the secondary effluent decreased, indicating that lower environmental temperatures (10–20 °C) may promote an elevation in the iARG concentration under chlorine exposure. Additionally, an NH4 concentration in the range from 1.8 to 2.5 mg/L also exhibited a significant impact on the iARG concentration, leading to an increase in the iARG concentration [67]. Huang et al., 2016, found that different pH levels during anaerobic sludge treatment not only influenced the community compositions of tetracycline-resistant bacteria (TRB) but also impacted their relative abundances, favoring the proliferation (under acidic pH conditions) or suppression (under alkaline pH conditions) of TRB [73]. Further analysis revealed that acidic pH conditions significantly enhanced both the quantity and genetic capabilities of key genetic elements responsible for the horizontal transfer of tetracycline ARGs, whereas alkaline pH conditions restricted their presence and activity. In addition, Li et al., 2018, conducted a redundancy analysis (RDA) to investigate the relationships among ARGs, mobile elements (*int*1 and *int*2), and external factors, such as tetracyclines (TC), sulfonamides (SA), quinolones (QN), macrolides (MC), the pH, the dissolved oxygen (DO), the electrical conductivity (EC),the total organic carbon (TOC), and the total nitrogen (TN) [74].The findings revealed no significant correlations between the presence of antibiotics and ARGs, except for the *sul* genes, which exhibited a negative correlation with sulfonamides (SAs), indicating potential co-selection with other ARGs or factors. The *tetB*, *sul*1, *sul*2, and *gyrA* genes and 16S rRNA gene copies showed positive correlations with the pH and EC (*p* < 0.05). Additionally, the abundance of the *tetW*, *sul*1, and *sul*2 genes correlated with the 16S rRNA gene, suggesting that bacteria in wastewater played a crucial role in the proliferation of the *tetW* and *sul* genes. Furthermore, the *sul*1 and *sul*2 genes exhibited significant correlations with the TOC (*p* < 0.05), indicating that a high TOC content likely contributed to bacterial reproduction. *TetW* was also positively correlated with the TN and DO (*p* < 0.05). Wu et al. explored how PAC and ferric chloride (FeCl_3_) affected the reduction in the ARB in secondary wastewater [75]. Coagulation effectively reduced four types of ARB (clindamycin-, sulfamethoxazole-, tetracycline-, and ciprofloxacin-resistant bacteria) by 37% to 95%. Despite this initial reduction, the abundance of ARB significantly increased in the reclaimed effluent after 4 days. The use of PAC and FeCl_3_ notably boosted the frequency of bacterial conjugation, the process where bacteria exchange genetic material, including ARGs. Factors like proper mixing, extended incubation, and an optimal temperature during coagulation enhanced this gene transfer. Larger bacterial aggregates formed during coagulation promoted closer cell interactions, facilitating gene exchange. Additionally, PAC and FeCl_3_ regulated the expression of genes related to conjugation, increasing the efficiency of genetic material transfer. The findings indicated that while coagulation with PAC and FeCl_3_ reduces the ARB initially, it could potentially accelerate the ARG spread during tertiary wastewater treatment, necessitating careful management and follow-up processes [75]. Furthermore, Johansson MHK et al., in their research, indicated that multiple factors play a role in the network responsible for disseminating ARGs [76]. They observed that climate conditions, or factors similar to the climate, influence the types of MGEs present in specific geographic regions. This, in turn, restricts the availability of MGEs accessible to bacteria and the potential interactions between different MGEs. Additionally, they discovered that certain MGEs have a wider range of host compatibility than others, which may enhance their ability to transfer genes across diverse bacterial species. These collective factors act as constraints on the potential pathways for gene transposition [76].

### 2.8. The Mechanisms Through Which Genetic Material Is Exchanged Between Clinical and Environmental Bacteria in WWTPs

Comprehending the mechanisms through which genetic material is exchanged between clinical and environmental bacteria is essential for managing the dissemination of AMR. Many ARGs are located on MGEs, such as plasmids, transposons, and integron-specific gene cassettes, acting as vehicles for these determinants and enabling their dissemination. HGT facilitates the transfer of genetic material between distinct species or even across different domains of life. It serves as a significant catalyst for microbial evolution, enabling the rapid acquisition of novel traits such as antibiotic resistance. This exchange of genetic material empowers microorganisms to effectively adapt to varying environmental conditions and evolutionary pressures. This adaptability becomes especially pertinent when confronted with environmental stressors and shifting circumstances. There are three primary mechanisms of HGT: transformation (direct DNA uptake), transduction (virally mediated transduction), and pili-mediated conjugation. Transformation entails the absorption of free DNA from the surrounding environment, transduction is facilitated by bacteriophages, and conjugation involves the direct transfer of genetic material through direct cell-to-cell contact. Recent findings have indicated that bacteriophages have a significant impact on HGT and recombination. Significant proportions of clinically relevant carbapenemase genes (*blaCTX-M*, *blaKPC*, *blaOXA-*48*-like*, and *blaNDM-*1) were detected in phage fractions isolated from environmental water. This indicates that bacteriophage transduction could potentially play a role in HGT. The study revealed a notable disparity in the prevalence of ARGs between the bacteriophage and bacterial fractions of water samples. ARGs were detected in 7.3% to 64.9% of the phage samples, whereas only 5.4% to 36.8% of the bacterial samples tested positive. Among the ARGs identified, *blaOXA-*48*-like* and *blaTEM* were the most widespread, occurring in 57.3% and 64.0% of the samples, respectively, across all sampling locations. These findings underscore the significant role of bacteriophages as reservoirs for ARGs and imply that phage-mediated transduction could be a crucial mechanism in the HGT of resistance traits among microbial communities [77]. Phage-mediated transduction is a significant mechanism facilitating HGT, as evidenced by metagenomic studies detecting a substantial proportion of bacterial genes within viral DNA fractions [78]. Using a network-based approach, it was demonstrated that *staphylococcal* hosts from different species, ecosystems, or antibiotic resistance phenotypes were closely interconnected through numerous phages. Additionally, it was revealed that the phages facilitating these connections had the ability to incorporate foreign genetic material, as indicated by the presence of a chloramphenicol resistance gene. These findings challenge the commonly reported host specialization of phages and highlight phages as potent vehicles for bacterial genetic exchange [79]. Phages, especially those with broad host ranges, can serve as reservoirs and carriers for antibiotic resistance, potentially aiding its spread in the environment. In one study, the majority of phages exhibited the ability to infect a wide variety of STEC strains, highlighting their broad host specificity. Notably, twelve *E. coli* phages were capable of infecting all four tested *E. coli* O157 strains. Among these, the *blaTEM* gene was detected in fifteen phages, and six of them were found to transfer *blaTEM* into *E. coli* ATCC 13706 via transduction. This widespread presence of ARGs has significant ecological implications, as it could promote extensive genetic exchange among bacterial hosts. Furthermore, the observation that three out of six transducing phages successfully transferred *blaTEM* genes across different serotypes provides further evidence of antibiotic resistance transfer facilitated by phages originating from the environment [80]. Although rare, certain transducing phages containing ARGs likely arise due to errors in packaging during the infection process of ARB. Some undegraded ARGs are released from ARB along with progeny phages, which may then be assimilated by other bacteria. This raises concerns about the potential for the horizontal transfer of ARGs facilitated by such phages. The *E. coli* strain *XX*13, resistant to multiple antibiotics (at least eight antibiotics), along with its associated phage (*YZ1*), was isolated from a municipal wastewater treatment facility. It is worth noting that although transducing phage particles carried some ARGs, the actual transduction process might be influenced by various environmental factors, such as the concentrations of the host bacteria and phages, temperature fluctuations, the presence of other substances in the medium, or other unknown factors [81]. Wastewater environments are hotspots for the selection of conserved chromosomal structures and diverse, adaptable plasmids. These conditions promote extensive gene exchange between plasmids, leading to the widespread dissemination of ARGs. A significant factor in this process is the high diversity of plasmids found in WWTP isolates, which correlates with an increased variety of ARGs. This diversity facilitates the proliferation of conserved *E. coli* STs and their specific associations with plasmids, driven by the continuous introduction of new resistance plasmids and the varying environmental conditions in WWTPs. These dynamic interactions enhance the exchange of genetic material, perpetuating the persistence and spread of ARGs within microbial communities in wastewater [82]. Bacteria from the *Aeromonadaceae*, *Moraxellaceae*, and *Bacteroidetes* families act as crucial reservoirs of antibiotic resistance in WWTPs. Specifically, *Aeromonadaceae* bacteria may play a significant role in the spread of antibiotic resistance within these systems. Additionally, *IncQ* plasmids and class 1 integrons, noted for their broad host range, have been highlighted as key vectors for HGT [83]. It has been observed that specific erythromycin ARGs can be efficiently transmitted between bacteria via MGEs like plasmids and transposons, resulting in the extensive spread of these resistance traits within the environment [84]. The discovery of plasmid-mediated genes responsible for beta-lactam resistance in nearly 10% of bacteria present in the final effluents of WWTPs and in over 32% of bacteria in the vicinity of WWTP areas confirms the dissemination of these genes into the environment, facilitating their further transmission among environmental bacteria. Moreover, the ability of these genes to transfer to an *E. coli* recipient strain indicates a significant potential for HGT among strains of different genera within sewage and environmental samples. All examined *bla* genes were transferable to *E. coli J*53 *(RifR)* through conjugation assays, indicating their location on transferable elements irrespective of their class. The frequency of conjugation ranged from 1.0 × 10^−5^ to 3.5 × 10^−5^ per donor strain, suggesting a substantial potential for HGT among strains of different genera within both sewage and environmental samples [85]. Continuous discharges of TWW exert a significant influence on a river’s resistome, impacting not only the quantity and diversity of ARGs but also facilitating their potential spread by enriching the river’s mobilome with a diverse range of MGEs. In all cases examined, downstream metagenomes exhibited notably elevated levels of sequences associated with both the phage integrase family (*PFAM no. PF00*589) and transposases, with more pronounced variations observed among sequences linked to the transposase 7 family (*PFAM no. PF0*1526), which includes transposases from *Tn*3, *Tn*21, *Tn*1721, *Tn*2501, and *Tn*3926. The substantial abundance and diversity of various integrase and transposase genes in downstream metagenomes strongly suggest that HGT likely plays a significant role in the acquisition and dissemination of ARGs in both the source UWWTP and the surrounding environment [86]. Simultaneously, Munke et al. highlighted that the overall dissemination of ARGs from WWTPs is comparable to that observed in soil environments [87]. Their analysis revealed a significant correlation (*p* < 0.001, based on 999 permutations) between the resistome of WWTPs and their microbial phylogeny, indicating a close association between the core resistome of WWTPs and the microbial community. Through sequencing the 16S rRNA of longitudinal samples collected over a 7-year period from the same WWTP, they observed remarkable stability in the microbial community within the WWTP, suggesting that the exchange of the WWTP resistome occurred to a limited extent within the WWTP environment [87]. Effluents from WWTPs have the potential to facilitate the HGT of MGEs and MDR phenotypes. Notably, a statistically significant correlation was found between the presence of integrons in *E. coli* isolates and resistance to fluoroquinolones, trimethoprim/sulfamethoxazole, amoxicillin/clavulanate, and piperacillin/tazobactam [88]. Despite the wastewater treatment process effectively reducing the concentration of integrons, their levels remained substantial (ranging from 10^5^ to 10^6^ copies/mL), with significant diversity in the effluents. This highlights the potential risks associated with the integron-mediated dissemination of ARGs into downstream environments. Among the three classes of integrons studied, class 1 integrons were found to be the most prevalent, consistent with previous research [89,90]. In contrast, the concentrations of class 2 and 3 integrons were relatively low compared to class 1 integrons. Additionally, class 1 integron gene cassettes were observed to carry more ARGs than class 3 integron gene cassettes, indicating that class 1 integrons may play a primary role in the spread of integron-mediated AMR. In activated sludge, where most ARG cassettes were located, class 3 integron gene cassettes exhibited greater diversity. This suggests that activated sludge may offer favorable conditions for the enhanced exchange and recombination of ARG cassettes within class 3 integrons [91]. A positive correlation was found between the increasing number of MDR strains and the presence of class 1 integrons, either individually or in conjunction with class 2 integrons, suggesting the involvement of integrons in facilitating multiple-antibiotic resistance. Additionally, the prevalence and diversity of *sul* and *dfr* genes indicated redundancy among plasmid-mediated sulfamethoxazole- and trimethoprim-resistant genes. These findings collectively underscore the significance of transmissible plasmid-mediated and integron-associated ARGs, specifically those conferring resistance to sulfamethoxazole and/or trimethoprim, among bacteria. This diversity potentially contributes to the persistence and dissemination of AMR [92]. Promiscuous, broad-host-range plasmids from the P incompatibility (*IncP*) group have been frequently implicated in the emergence and dissemination of ARGs among Gram-negative bacteria. These versatile plasmids facilitate the transfer of resistance determinants across diverse bacterial species, significantly contributing to the spread of AMR. The combined action of an integron and the conjugative DNA transfer modules of an *IncP-*1 broad-host-range plasmid is crucial for the rapid emergence and spread of enhanced resistance traits. It has been shown that an integron on the *IncP-*1*β* multi-resistance plasmid, specifically *pB*10, can function by integrating an additional resistance gene cassette into the central segment of the integron. Moreover, the insertion of an *IS*10 element can lead to increased levels of β-lactam resistance [93]. The presence of genes for ESBL, integron integrase, AMR, and MDR efflux in bacteria isolated from municipal WWTPs suggests that these facilities’ wastewater may be a critical area for the development and spread of MDR bacteria. This situation could pose significant risks to both human health and the environment [94]. In addition to HGT and the selection of resistant organisms/genetic determinants, the preferential elimination of susceptible organisms compared to their resistant counterparts can also play a significant role in the increase in antimicrobial resistance. Notably, certain subsets of *E. coli*, such as type *A*0, which were selectively removed during wastewater treatment, were found to contribute to the observed increase in the AMR prevalence in the final effluent [95]. Abdulkadir et al.’s analyses indicated that only 10.26% of the annotated ARGs were located on plasmids, suggesting that the majority of ARGs were not transmitted through plasmids [96]. Most of the annotated ARGs were found on chromosomes, indicating that these genes were primarily passed down through vertical gene transfer (VGT), from parent to offspring. While ARGs on plasmids are prone to be transferred between different bacterial species, chromosome-located ARGs are the main contributors to the spread of ARGs within bacterial communities [96]. In addition, Meng et al. (2017) evaluated five ARGs and found that none exhibited a significant positive correlation with *intI1* (*p* < 0.05) over the operational period [97]. This indicates that HGT pathways may not have been the primary mechanism for the spread of ARGs in the system. Instead, it suggests that VGT played a major role in shaping the ARG profiles observed [97]. Amirsoleimani et al. (2021) emphasized that out of the ten plasmids examined in their study, five originated and were exclusively carried within a single species or clonal complex of a species [98]. The exclusive presence of these plasmids within a clonal complex of *S. aureus* or a *staphylococcus* species suggests their capacity for conservation within a specific group, with higher coverage levels indicating more recent acquisition and greater preservation. This pattern also suggests VGT [98].

## 3. Discussion

The studies reviewed highlight the significant role of wastewater and aquatic environments as critical reservoirs and conduits for the dissemination of ARGs. The selection pressure from antibiotics in wastewater can significantly impact the resistance of environmental bacteria. The main findings reveal the pathways and mechanisms of ARG dissemination, supported by extensive datasets and the consistent detection of clinically relevant ARGs at diverse wastewater sites and sources. Strong correlations have been highlighted between the residual concentrations of antibiotics, such as ciprofloxacin and carbapenems (e.g., meropenem), and the presence of ARGs like *blaNDM* and *blaOXA48*. Moreover, ARGs such as *blaKPC*, *blaNDM*, and *mcr-*1 persist in various wastewater sources, with particularly high frequencies in hospital and veterinary wastewater. This underscores the critical role of clinical environments in amplifying the risks of AMR. Numerous studies have confirmed the high prevalence of ARGs in untreated urban wastewater and treated wastewater. For example, bacteria such as *Aeromonas* spp. and *Enterobacterales* harbor resistance mechanisms, including beta-lactamases and plasmid-mediated quinolone resistance genes, with some genes (*qnrS*2, *aac(*6*′)-Ib-cr*) persisting even after treatment. These findings emphasize the urgent need for targeted strategies to address the public health challenge posed by AMR [17,18,19,36]. The inability of WWTPs to completely eliminate ARGs represents a significant threat, turning treated environments into hotspots for genetic exchange among bacteria and amplifying resistance [20,21]. WWTPs exhibit a vast genetic diversity, largely linked to plasmids and MGEs, which facilitate gene exchange among bacteria. Integrons, particularly class 1 integrons, play a central role in this process, acting as vectors for ARGs. They not only carry a single gene but often transport multiple genes, increasing the complexity of the phenomenon. Other key players in this network of dissemination are bacteriophages. These phages not only act as reservoirs of ARGs but also transfer these ARGs to new bacterial hosts through transduction, even in environmental settings such as rivers. This means that the treated effluent, released into aquatic ecosystems, serves as a vehicle for resistance dissemination, enriching environmental resistomes [77,78,79,80]. A particularly concerning scenario arises from pharmaceutical effluents, which contain high concentrations of antibiotics. These effluents alter environmental microbial communities, favor the selection of ARB, and amplify the presence of ARGs. For instance, carbapenemase genes, such as *blaNDM-*1 and *blaOXA-*23, have been detected in treated effluents and are often genetically linked to clinical isolates, indicating a dangerous connection between the environment and human infections. Downstream of pharmaceutical discharges, river sediments become reservoirs of ARGs [20,21,23,24]. Studies have shown that some genes, such as those associated with macrolides, persist even kilometers away from the source [23,24]. This phenomenon underscores the selective pressure exerted by anthropogenic pollution, which enhances the genetic mobility among bacteria and accelerates HGT [20,21]. Human activities, therefore, amplify the diversity of ARGs in wastewater. Contamination promotes the absorption of these genes by microbial communities, fostering genetic exchange processes both in laboratory settings and field experiments. This has direct implications not only for ecosystems but also for wildlife and human health, as ARB propagate in the environment and may eventually return to us through the food chain or other exposure pathways [26]. These studies highlight that current WWTP methods are insufficient for effectively removing antibiotics, ARB, and ARGs, particularly in pharmaceutical and hospital effluents. Therefore, advanced treatment technologies, stricter discharge regulations, and the regular monitoring of ARGs in aquatic environments are necessary to understand their dynamics and implement timely interventions. Biological treatment, although effective in reducing certain categories of resistance, can create conditions conducive to the proliferation of bacteria with higher levels of resistance, necessitating vigilant monitoring to prevent such phenomena. The findings suggest that environmental management strategies, such as optimized bioreactor systems and controlled antibiotic usage, can mitigate the proliferation of ARGs [20,35,36,45,51,55,67,73,75,76,99]. Advanced technologies such as MBRs demonstrate considerable potential for improving the water quality but come with the trade-off of an increased risk of HGT, which could exacerbate the resistance issue. This phenomenon is driven by the high bacterial density and microbial diversity present in the treatment environment, which foster ideal conditions for the transfer of ARGs among bacteria. The presence of transconjugants, which can act as secondary donors, further aggravates the problem [54]. These findings underscore the critical need to complement traditional treatments with integrated strategies aimed at curbing the spread of ARB and ARGs. Measures such as enhanced disinfection processes, the continuous monitoring of antibiotic residues, and the implementation of additional barriers against genetic transfer should be prioritized. The enrichment of ARGs is linked to microbial metabolic activities, highlighting how various environmental factors contribute to the persistence and amplification of resistance [48,49,50,51,52]. For example, the diversity of bacterial communities within biofilms plays a critical role in shaping the resistome. Biofilms serve as both sources and reservoirs of ARB and act as highly efficient sites for HGT. These findings underscore the urgent need to develop targeted strategies to manage biofilm formation and reduce the persistence of ARGs in wastewater systems, as well as to control the spread of AMR in these environments to protect public health [50,51,52].

In addition, the persistence and proliferation of ARGs in WWTPs are influenced by a complex interplay of environmental, chemical, and operational factors. Key drivers include water quality parameters such as the pH, conductivity, TN, ammonium levels, and CODMn, which show significant correlations with specific ARGs. Notably, the temperature plays a nuanced role: while not directly linked to the overall ARG abundance, specific ranges (e.g., 25–27 °C) enhance eARGs, whereas cooler temperatures (10–20 °C) promote iARGs under chlorine exposure [67,73]. The pH of the water is particularly critical. Acidic conditions foster the proliferation of ARB (e.g., TRB) and enhance the HGT capabilities, while alkaline conditions suppress these processes. The nutrient availability, especially that of TOC and TN, further supports bacterial growth and ARG dissemination, underscoring the role of organic matter in amplifying resistance mechanisms [74].

Similarly, the presence of antibacterial residues, heavy metals, and non-therapeutic agents like TCS play significant roles in the development of resistance. Zn, Pb, and heavy metals in general serve as persistent selective pressures, fostering the growth of ARGs in microbial communities. For instance, significant correlations between the presence of Zn and ARGs highlight its role in enhancing resistance. In contrast, the presence of Cr and Cu showed weaker or no significant correlations with ARGs [40]. Additionally, the presence of TCS, often used as an antibacterial agent in consumer products, had a strong correlation with ARGs, particularly in wastewater, suggesting its critical role as a selecting agent. The use of biocides like BC, which could replace TCS in many products, further adds to the problem by leading to cross-resistance, particularly to last-resort antibiotics like colistin [48,49].

This makes managing resistance a multifaceted challenge. The One Health approach, which emphasizes the interconnectedness of human, animal, and environmental health, should guide the development of integrated policies that consider all sources of AMR, including wastewater. Only through a holistic and sustainable approach can the risks posed by AMR be effectively mitigated, ensuring the protection of both the environmental integrity and public health.

## 4. Materials and Methods

### 4.1. Study Design

This scoping review was conducted according to the preferred reporting items for systematic reviews and meta-analyses extension for scoping reviews (PRISMA-ScR) protocol. This approach was particularly suitable for mapping the broad issue of the AMR spread via wastewater, allowing for the identification of key concepts, gaps, and a wide range of evidence types. To ensure comprehensive coverage of relevant studies, the review included research that examined wastewater as a source of AMR, encompassing hospital effluents, IWW, urban sewage, and agricultural runoff. The review focused on studies that explored the pathways through which AMR spreads and the factors that influence this process. There were no geographical restrictions, and both quantitative and qualitative empirical research were included. Studies that focused solely on AMR without any mention of wastewater and/or WWTPs were excluded, as were articles not available in English.

### 4.2. Search Strategy

The search for the relevant literature published up to June 2024 was conducted across the PubMed and Web of Science databases. Additional studies were identified through citation chasing, using the reference lists of selected articles to find further relevant research. The search strategy was designed with the following combination of key terms: ((Drug Resistance) OR (Antibiotic Resistance) OR (Antimicrobial resistance) OR (Antibacterial Resistance)) AND (Wastewater OR (Waste Water*)).

### 4.3. Study Selection

All retrieved studies were imported into reference management software (i.e., EndNote) to remove duplicates. Two independent reviewers then screened the titles and abstracts to evaluate their relevance according to the predefined inclusion and exclusion criteria. In cases of disagreement, a third reviewer was consulted to resolve any inconsistencies.

### 4.4. Data Extraction and Synthesis

A data extraction form was developed to collect key information, including the study characteristics (authors, year, location, study design), identified AMR pathways, influencing factors, proposed or studied interventions, and study outcomes related to the spread of AMR. The data were then synthesized thematically to provide a structured understanding of the findings.

## 5. Conclusions

In an effort to minimize the incidence and spread of AMR and the associated risks to human and animal health, it is crucial to identify the hotspots and transmission pathways of ARGs. Grasping the role of MGEs in the development of antibiotic resistance is crucial for efforts to curb the spread of ARGs. Understanding how these genes are transferred and the mechanism behind their movement is key to formulating strategies to reduce the spread of antibiotic resistance in both healthcare and environmental contexts. In this context, WWTPs are recognized as one of the main sources of ARB and ARGs in aquatic environments. Therefore, the aim of this scoping review was to assess the impact of wastewater and WWTPs on promoting the evolution and emergence of resistance in antimicrobial-contaminated environments and to gather information on the transmission of ARGs in WWTPs to develop effective strategies for controlling antibiotic resistance in the environment. High growth rates and dense microbial populations, which are essential for traditional biological treatments in WWTPs, combined with the presence of organic and inorganic nutrients and an optimal temperature and pH, create an extremely conducive environment for the transfer of ARGs and the emergence of multi-resistance among bacteria [20,35,36,45,51,55,67,73,75,76,99]. Additionally, the potential for the co-selection of ARGs increases when bacterial communities are exposed to chemical stress (such as heavy metals, antibiotics, or both) in the constantly changing environment of a WWTP [40,41,47]. Furthermore, the lack of adequate technologies in wastewater treatment contributes to the release of a high number of ARB and ARGs in the final effluents. Despite the findings highlighting the urgency of implementing more effective mitigation strategies, several critical issues emerged that require further attention. Firstly, many conventional treatments fail to completely eliminate ARGs, and disinfection processes, if poorly calibrated, may even promote the selection of more resistant strains. For example, UV irradiation and chlorine use as standalone processes can be effective; however, to ensure the maximum efficacy, modifications such as the combined use of both processes, the integration of oxidizing agents, or specific pretreatments might be necessary [59,60,61,70]. There is a need for a better understanding and assessment of the risks to human health and ecosystems posed by the presence of ARB and ARGs in WWTPs and in the context of wastewater reuse. Additionally, it is essential to comprehend how technological interventions would impact the overall effectiveness of treatment and the economic feasibility of wastewater reuse applications. From a critical perspective, it is important to highlight several gaps in the studies reviewed. Most research focused on specific types of antibiotics or environments, which limits the generalizability of the findings. Moreover, the ecological and health impacts of the interactions between ARGs and environmental factors remain largely underexplored. For instance, the role of MGEs and biofilms in resistance transmission warrants deeper investigation to elucidate the HGT mechanisms in varied environmental conditions. Another critical issue is the lack of standardization in protocols for monitoring ARB and ARGs, which make direct comparisons between studies difficult and hinder the evaluation of the treatment efficacy. This inconsistency underscores the need for harmonized methodologies to ensure reliable and actionable data.

In conclusion, effectively tackling the global challenge of AMR requires the integration of interdisciplinary approaches and One Health-based solutions. Future research should prioritize the development of policies that incentivize systematic monitoring and the adoption of advanced technologies. Reducing the risks of AMR and safeguarding public health and the environment will only be possible through collective efforts and a stronger focus on local contexts.

## Figures and Tables

**Table 1:** Summary of studies investigating the impact of wastewater treatment mechanisms on AMR dynamics.

Treatment	Effects on ARB/ARGs	Additional Notes	References
Biological treatment (secondary treatment)	-Low resistance to erythromycin unchanged (70% survival) -Medium resistance decreased (from 18% to 6%, *p* = 0.007) -High resistance proliferated (from 0.2% to 10%, *p* = 0.011) with increased ARB diversity	Environment favorable to ARB proliferation Resistance amplified during activated sludge treatment	[53]
Membrane bioreactor (MBR) (advanced treatment)	-The abundance of the RP4 plasmid, a model for studying gene transfer, in total DNA remained high and stable at 10^4^ copies per milligram of biosolids, indicating that ARGs persisted in the system and the facilitation of the HGT of plasmids (e.g., RP4) -Transconjugators acted as secondary donors	There was high bacterial density, microbial diversity, and nutrient richness; the study highlights the potential for ARGs to spread within MBRs, posing a health risk due to the persistence and dissemination of these genes in wastewater treatment systems	[54]
Nitrification/denitrification and clarification processes (secondary treatment)	-A common core group of ARGs was found in all WWTP sections; these genes not only persisted but showed higher transcription levels in effluents than in influents, indicating that treatment did not completely eliminate them -ARGs and MGEs showed significantly higher transcriptional activity in effluents compared to biological reactors -Effluents released actively transcribed ARGs, including genes associated with human pathogens, suggesting a risk to public and environmental health	Improving the settleability of sludge in secondary clarifiers or implementing membrane filtration could reduce the release of ARGs in effluents	[55]
Pretreatments (ultrasonification, alkaline hydrolysis, and alkaline ultrasonification) + anaerobic digestion of sludge (secondary treatment)	-Reduction in ARB abundance in sludge by up to 90% -Increased the risk of ARB spreading in the supernatant	Pretreatments increased the abundance of some ARB in the supernatant (e.g., tetracycline ARB increased from 0.6 × 10^2^ CFU/mL (direct digestion) to 2.3 × 10^2^ CFU/mL after pretreatments); it is essential to pay attention to the treatment of the supernatant to mitigate the ecological risks associated with the spread of AMR	[56]
Suspended growth system vs. biofilm-based system (secondary treatment)	-Both WWTPs exhibited a reduction of over 90% in ARGs per genome equivalent -Despite the reduction in the abundance, the proportion of ARGs associated with MGEs increased significantly from the influent to the effluent in each plant; strong positive correlations were observed between ARGs and Antibiotic Production Genes (APGs) for specific antibiotic classes in both treatment systems -The biofilm system exhibited a higher diversity and persistence of ARGs during the secondary treatment process compared to the suspended growth system		[57]
SBR (sequential batch reactor): anaerobic reactors (secondary treatment)	-Significant increase in certain ARGs, such as those associated with tetracycline and beta-lactam resistance -Promoted ARB proliferation: enrichment of microbial taxa known to host ARGs	It has the capacity to degrade antibiotics, but it is not sufficient to completely eliminate the selective pressure	[58]
Ultraviolet disinfection (UV) (tertiary treatment)	-Reduction in viable and cultivable bacteria, but persistence of bacterial DNA (including ARGs) -ARGs located on MGEs facilitated diffusion even in the absence of viable bacteria -Reduction of about 50% for blaCTX-M and intI1; no reduction for qnrB -ARG detection rate in reuse water varied between 0% and 16.7% -Significant reductions in bacterial loads were achieved from the secondary to the tertiary treatment phase; the relative abundance of ARGs increased in the reuse water compared to the secondary effluent	Highlights the need for advanced strategies to degrade resistant genetic material	[19]
Secondary treatment vs. ultraviolet disinfection (UV) (tertiary treatment)	The secondary treatment led to the most pronounced variations in the bacterial community composition, accompanied by a significant reduction of approximately 2 log units in the abundance of ARGs. In contrast, the UV disinfection of the secondary effluent did not result in notable changes in the bacterial community structure or ARG abundance. However, UV treatment did lead to a reduction in the viability of culturable enterobacteria, with a loss of around 2 log units.		[59]
UV disinfection + chlorination (tertiary treatment)	-UV disinfection/chlorine (320 mJ/cm^2^ + 2 mg/L) increased ARG reduction by 1–1.5 log compared to UV disinfection alone: UV disinfection/chlorine (UV dose ≥ 4 mJ/cm^2^, chlorine concentration ≥ 1 mg/L) caused synergistic effect and decrease in RP4 plasmid transfer -UV disinfection/chlorine suppressed photoreactivation and increased removal efficiency	-The efficacy of disinfection was influenced by the specific type of resistance and the bacterial characteristics -Chlorine at low doses (<2 mg/L) increased HGT, highlighting ecological risks	[60]
Chlorination (tertiary treatment)	-Gram-negative bacteria (e.g., *E. coli*) were less tolerant than Gram-positive bacteria (e.g., Enterococcus) -Effective reduction in bacterial load, but incomplete elimination of ARGs and MGEs		[61]
Chlorination (tertiary treatment)	-Promoted the selection of chlorine-resistant pathogenic bacteria and the regrowth of such bacteria in reclaimed water with long retention times -Reactivation and regrowth of bacteria were most likely to occur after exposure to lower chlorine doses, with reactivation decreasing gradually as chlorine dose increased -Chlorine selectivity promoted resistance in Salmonella		[62]
Secondary treatment and chlorination (tertiary treatment)	-Secondary treatment raised the relative abundance of ARGs in the remaining bacteria; disinfection effectively reduced this abundance -Low-dose chlorination (<8 mg/L) may increase AMR via HGT		[63]
Chlorination and ultraviolet disinfection (tertiary treatment)	-Sublethal doses of these disinfection methods led to an increased expression of the tetA gene, potentially enhancing tetracycline resistance -Chlorine concentrations exceeding 1.0 mg Cl2/L and a contact time of 10 min increased the tetracycline resistance in *E. coli*	Disinfection processes are essential for eliminating pathogenic bacteria, but inadequate dosing may inadvertently promote antibiotic resistance	[64]
Chlorination (tertiary treatment)	Chlorine selectivity promoted resistance in Pseudomonas		[65]
Chlorination (tertiary treatment)	The resistance rates to TET, sulfamethoxazole, chloramphenicol, streptomycin, ampicillin, and gentamicin of Salmonella isolated from the effluent after chlorination exhibited a significant increase compared to those before chlorination in a WWTP		[66]
Chlorination (tertiary treatment)	-Increased intracellular antibiotic resistance genes (iARGs) and extracellular antibiotic resistance genes (eARGs) -Increased concentration of tetA, tetB, sul3, ampC, aph(2′)-Id, and vanA		[67]
Ultrafiltration and ozonation (tertiary treatment)	-Ozonation was effective in reducing the concentrations of exDNA and associated ARGs; however, the effectiveness of the process depended on several factors, including the dose of ozone applied and the specific characteristics of the treated wastewater -Ultrafiltrated effluents showed exDNA concentrations that had been reduced to 0.7 ± 0.1 ng/mL; despite these reductions, exDNA accounted for up to 80.0 ± 5.8% of the total DNA in treated effluents		[68]
Comparison between conventional activated sludge (CAS) process, upflow anaerobic sludge blanket (UASB) reactor followed by biological trickling filter (BTF) (primary and secondary treatment), and modified activated sludge process followed by UV disinfection (MAS/UV)	-The MAS/UV system was more effective in reducing the ARB than the other two systems studied (MAS/UV: 2–3 log reduction; CAS: 1 log reduction; UASB/BTF: 0.5 log reduction) -Composition of ARB: raw wastewater: predominance of Escherichia (56 out of 118 isolates); treated effluent: Escherichia (31 out of 118), followed by Bacillus (22 out of 118), Shigella (14 out of 118), and Enterococcus (14 out of 118)		[69]

## Data Availability

No new data were created or analyzed in this study. Data sharing is not applicable to this article.
